# Diversification of the Genus *Anopheles* and a Neotropical Clade from the Late Cretaceous

**DOI:** 10.1371/journal.pone.0134462

**Published:** 2015-08-05

**Authors:** Lucas A. Freitas, Claudia A. M. Russo, Carolina M. Voloch, Olívio C. F. Mutaquiha, Lucas P. Marques, Carlos G. Schrago

**Affiliations:** Departamento de Genética, Universidade Federal do Rio de Janeiro, RJ, Brazil; Virginia Tech, UNITED STATES

## Abstract

The *Anopheles* genus is a member of the Culicidae family and consists of approximately 460 recognized species. The genus is composed of 7 subgenera with diverse geographical distributions. Despite its huge medical importance, a consensus has not been reached on the phylogenetic relationships among *Anopheles* subgenera. We assembled a comprehensive dataset comprising the COI, COII and 5.8S rRNA genes and used maximum likelihood and Bayesian inference to estimate the phylogeny and divergence times of six out of the seven *Anopheles* subgenera. Our analysis reveals a monophyletic group composed of the three exclusively Neotropical subgenera, *Stethomyia*, *Kerteszia* and *Nyssorhynchus*, which began to diversify in the Late Cretaceous, at approximately 90 Ma. The inferred age of the last common ancestor of the *Anopheles* genus was ca. 110 Ma. The monophyly of all *Anopheles* subgenera was supported, although we failed to recover a significant level of statistical support for the monophyly of the *Anopheles* genus. The ages of the last common ancestors of the Neotropical clade and the *Anopheles* and *Cellia* subgenera were inferred to be at the Late Cretaceous (ca. 90 Ma). Our analysis failed to statistically support the monophyly of the *Anopheles* genus because of an unresolved polytomy between *Bironella* and *A*. *squamifemur*.

## Introduction

Malaria is a vector-borne disease that is transmitted by *Anopheles* mosquitoes infected with *Plasmodium* protozoans. Although declining in incidence, 207 million cases of this tropical malady were estimated in 2012, with more than 620,000 casualties; most of these were in the African continent (www.who.int/gho/malaria). The decline has been associated with the implementation of effective protective measures for exposed individuals and with a reduction in the longevity and density of the vector mosquito population (WHO, World Malaria Report 2012).

The mosquito genus *Anopheles* belongs to the subfamily Anophelinae, family Culicidae [1], a monophyletic group supported by molecular phylogenetics [2, 3] and numerous distinct morphological features [4]. The genus currently harbors 465 recognized species that are allocated across seven subgenera based on the number and position of the specialized setae on the male genitalia. Nevertheless, the diversity and the geographical distribution of the species assigned to each *Anopheles* subgenus vary tremendously [1].

The two largest subgenera, for instance, include 86% of the diversity of the genus *Anopheles*: the cosmopolitan *Anopheles* with 182 species and the Old-World *Cellia* with 220 species. Four smaller subgenera are restricted to the Neotropics: *Kerteszia* with 12 species, *Lophopodomyia* with six species, *Nyssorhynchus* with 39 species and *Stethomyia* with five species. Finally, the monotypic *Baimaia* subgenus has only recently been described to include the type species *A*. *kyondawensis*, which is restricted to the Oriental region [5]. The four largest subgenera contain clades of the 40 species that have been identified as dominant malaria vectors [6].

Molecular and morphological studies have generally supported the monophyletic status of the subgenera *Cellia*, *Nyssorhynchus* and *Kerteszia* [2–4]. Additionally, a close association has been consistently found between two neotropical subgenera, *Nyssorhynchus* and *Kerteszia* [3, 4, 7]. Nevertheless, the relationship between this neotropical clade with the other neotropical subgenera (*Lophopodomyia* and *Stethomyia*) has not been properly tested as many studies lacked the necessary taxon sampling [2, 3, 8].

One exception was the study of Sallum et al. [7], which used molecular markers that included all major *Anopheles* subgenera and approximately 30 Anophelinae species. Nevertheless, in that study, the monophyletic status of *Nyssorhynchus* was challenged for the first time as *Kerteszia* grouped within that clade. Additionally, the study indicated as tentative the placement of the neotropical *Stethomyia* (grouped to the non-tropical *Cellia* subgenus in the phylogeny) as this taxon was represented by one species sequenced for a single marker. Because the statistical support for the clades in the study was generally low, it remains to be determined whether a clearer picture emerges if taxon and marker sampling is incremented, particularly for the neotropical *Stethomyia*.

In this study, we used a comprehensive phylogeny with 50 species representing all major subgenera to unveil the neotropical diversification in the genus *Anopheles*. As the number and the time of colonizations to the continent are critical for a well-defined diversification picture, we also included time tree and ancestral area reconstruction analyses that allowed us to make biogeographical considerations regarding the colonization of the Neotropics by ancestral anophelines. Our most prominent result is the statistical support for a new clade composed of the subgenera *Stethomyia*, *Kerteszia* and *Nyssorhynchus* that is confined to the Neotropical region.

## Materials and Methods

### Taxon sampling and molecular markers

We included 47 *Anopheles* species representing six out of the seven subgenera. From each subgenus, all species that are considered to be dominant vectors for malaria were included in the dataset to refine their phylogenetic position. The classification of the species in subgenera followed [1]. As the monophyletic status of *Anopheles* has been previously questioned, we have also included species of the other two Anophelinae genera: *Chagasia bathana* and *Bironella hollandi*. The topology was rooted using *Aedes aegypti* sequences. Phylogenetic analyses were conducted using three molecular markers. Two markers were mitochondrial, the cytochrome oxidase subunits I and II (COI and COII), and one was nuclear, the ribosomal 5.8S subunit. Molecular markers were selected based on the availability of sequences for a large portion of the *Anopheles* diversity, avoiding an incomplete supermatrix. Sequence data were downloaded from GenBank, and accession numbers are provided in Table 1.

**Table 1:** GenBank accession numbers of sequences.

Subgenus	Species	*COI*	*COI*	*COII*	*5*.*8S*
*Anopheles*	*Anopheles atroparvus*	-	-	-	AY634533
*Anopheles*	*Anopheles barbirostris*	AY729982	EU797194	AB331589	EU812783
*Anopheles*	*Anopheles freeborni*	-	AF417717	AF417753	-
*Anopheles*	*Anopheles labranchiae*	HQ860331	-	-	AY365008
*Anopheles*	*Anopheles lesteri*	EU699001	-	EU699056	AF384172
*Anopheles*	*Anopheles messeae*	HE659586	-	AY953352	AF504213
*Anopheles*	*Anopheles pseudopunctipennis*	HM022407	AF417721	AF417757	U49735
*Anopheles*	*Anopheles quadrimaculatus*	NC_000875	NC_000875	NC_000875	U32503
*Anopheles*	*Anopheles sacharovi*	AY135694	-	-	AF462088
*Anopheles*	*Anopheles sinensis*	AY768950	HM488283	AF325715	HM590510
*Cellia*	*Anopheles aconitus*	DQ000253	-	AF194448	DQ000252
*Cellia*	*Anopheles annularis*	AY917197	-	EU620675	DQ478878
*Cellia*	*Anopheles arabiensis*	AF252877	AF417705	AF417741	DQ287723
*Cellia*	*Anopheles balabacensis*	-	-	U94289	-
*Cellia*	*Anopheles culicifacies*	-	AF116834	HQ377221	AY168883
*Cellia*	*Anopheles dirus*	-	AJ877310	AJ877309	U60410
*Cellia*	*Anopheles farauti*	HQ840792	HQ840792	DQ674709	HM584396
*Cellia*	*Anopheles flavirostris*	-	AY943650	AJ512742	GU062188
*Cellia*	*Anopheles fluviatilis*	GQ906980	AF116830	AJ512740	GQ857445
*Cellia*	*Anopheles funestus*	NC_008070	NC_008070	NC_008070	JN994135
*Cellia*	*Anopheles gambiae*	NC_002084	NC_002084	NC_002084	NW_163551
*Cellia*	*Anopheles koliensis*	HQ840838	HQ840838	U94304	EF042756
*Cellia*	*Anopheles latens*	-	DQ897936	-	-
*Cellia*	*Anopheles maculatus*	JN596972	-	AF448468	AY803346
*Cellia*	*Anopheles melas*	DQ792579	DQ792579	DQ792579	GQ870314
*Cellia*	*Anopheles merus*	-	-	-	GQ870313
*Cellia*	*Anopheles minimus*	GQ259180	AF116832	AF194452	DQ336436
*Cellia*	*Anopheles moucheti*	-	DQ069721	DQ069719	-
*Cellia*	*Anopheles nili*	-	DQ069722	DQ069720	-
*Cellia*	*Anopheles punctulatus*	HQ840857	HQ840857	U94312	HM584446
*Cellia*	*Anopheles sergentii*	-	-	-	AY533851
*Cellia*	*Anopheles stephensi*	FJ210893	AF417713	AY949851	EU847233
*Cellia*	*Anopheles subpictus*	AF222327	AF417711	EF601864	GQ870328
*Cellia*	*Anopheles sundaicus*	AF222324	AF417712	AF417748	AF369559
*Cellia*	*Anopheles superpictus*	-	AY900633	FJ526436	DQ487148
*Kerteszia*	*Anopheles bellator*	-	-	AF417740	DQ364652
*Kerteszia*	*Anopheles homunculus*	JQ291235	-	-	JQ291246
*Kerteszia*	*Anopheles lepidotus*	JQ041282	-	-	JN967765
*Lophopodomyia*	*Anopheles squamifemur*	-	AF417723	AF417759	-
*Nyssorhnchus*	*Anopheles albimanus*	-	AF417695	AF417731	L78065
*Nyssorhnchus*	*Anopheles albitarsis*	HQ335344	HQ335344	HQ335344	AF462385
*Nyssorhnchus*	*Anopheles aquasalis*	-	AF417697	AF417733	DQ020123
*Nyssorhnchus*	*Anopheles darlingi*	NC_014275	NC_014275	NC_014275	GU477277
*Nyssorhnchus*	*Anopheles marajoara*	DQ076216	DQ076216	AF417735	AY028127
*Nyssorhnchus*	*Anopheles nuneztovari*	AF368065	AF270915	AF417736	HQ020405
*Stethomyia*	*Anopheles acanthotorynus*	-	AF417724	AF417760	-
*Stethomyia*	*Anopheles nimbus*	HM022409	HM022409	-	-
Outgroup	*Aedes aegypti*	NC_010241	NC_010241	NC_010241	M95126
Outgroup	*Bironella hollandi*	-	-	EU477545	EF619445
Outgroup	*Chagasia bathana*	AF417726	AF417726	AF417762	-

### Alignment and phylogenetic analysis

Each gene was aligned individually using the program MAFFT 7 [9]. Alignments were then inspected and edited in MEGA version 5.1 [10]. Individual alignments were then concatenated in the SeaView 4 program [11] assuming species-level monophyly. The final alignment matrix included 157 bp of the 5.8S marker, 525 bp of the first segment of COI and 562 bp of the second segment, and 684 bp of COII, summing to a total length of 1,928 bp. The final alignment is available online at the Dryad database and at www.edarwin.net/data/Anopheles. Two methods of phylogenetic reconstruction were implemented using the GTR+G+I substitution model as indicated by the jModelTest2 program [12]. The first was a Bayesian inference (BI) method conducted in the program MrBayes 3.2 [13]. The Markov Chain Monte Carlo (MCMC) algorithm was executed in two independent runs. Each run was sampled every 1,000^th^ generation until 10,000 trees were obtained, with 25% excluded as burn-in. In this tree, the clade Bayesian posterior probability (BP) was used as a metric of topological support. Convergence of the chains was assessed via the potential scale reduction factor, which was close to 1.0 for all parameters, and the effective sample size (ESS), which was > 200 for all parameters. The second method was maximum likelihood (ML). In this case, the algorithm was implemented in the PhyML program package 3 [14], and the topological support test was the approximate likelihood ratio statistic, aLRT [15].

We have also investigated whether data partitioning would impact topological inference. Partitioning scheme was inferred with the PartitionFinder software [16] by searching through all substitution models and using the Bayesian information criterion (BIC) to choose between alternative models. Three data blocks were tested, namely, 5.8S, COI and COII, and the greedy search algorithm was used. The best partitioning scheme was composed of two partitions, a mitochondrial partition containing COI and COII, under the GTR+G+I model, and a single partition for 5.8S, under the K80+G model. Phylogenetic inference using the estimated partitioning scheme was conducted in MrBayes, using the same MCMC settings as above, and also in RAxML 8 [17], which implements a fast maximum likelihood topological search.

### Divergence times and ancestral area reconstruction analyses

The molecular dating analysis was conducted in a Bayesian framework with the program BEAST 1.7.8 [18] that also uses a MCMC algorithm to infer the posterior distribution of the parameters. As in the phylogenetic inference, the elected model for nucleotide substitution was GTR+G+I. The prior distribution of the evolutionary rates along branches was modeled by the uncorrelated lognormal distribution, whereas the Yule process was adopted to model the tree prior. The MCMC run consisted of 100,000,000 generations with parameters sampled every 1,000^th^ step. A burn-in period of 25,000 generations was discarded. The BEAST analysis was repeated twice to check for convergence, which was assessed by the potential scale reduction factor as implemented in the coda package of the R programming environment (www.r-project.org). ESSs were also calculated in Tracer 1.6, resulting in values > 200 for all parameters.

To decompose the branch lengths (i.e., genetic distances) into absolute times and evolutionary rates, calibration priors on node ages are required. Usually, these priors are obtained from the fossil record or from the mean evolutionary rate. As with most non-vertebrate taxa, however, the *Anopheles* fossil record is very scarce because only two *Anopheles* fossils are currently recognized. The oldest record is *Anopheles* (*Nyssorhynchus*) *dominicanus* from the Late Eocene (33.9–41.3 Ma) [19], and the most recent is *Anopheles rottensis* from the Late Oligocene (13.8–33.9 Ma) [20]. Nevertheless, the usage of these records as time priors has been deemed notably problematic. Although the *A*. *domincanus* fossil was assigned to subgenus *Nyssorhynchus*, the age of the fossil varies from 15 Ma to 45 Ma, depending on the dating technique applied [8]. Thus, as in many studies with mosquitoes, we have relied on the split between *Aedes* and *Anopheles* that has been estimated at 145 Ma (97.7–193.7) by Logue et al. [21], in which the timescale was calibrated using the estimate of the *Drosophila*-*Anopheles* divergence at 260 Ma obtained by Gaunt and Miles [22]. Thus, a Gaussian calibration prior with mean = 145 Ma and standard deviation = 25 Ma was adopted to accommodate the 97.7–193.7 range within the 95% highest probability density interval.

For the ancestral area reconstruction analysis, we first associated each terminal taxon to one of the following area categories: (1) Americas; (2) Africa; (3) Europe; (4) India plus West Asia; and (5) Southeast Asia plus the Pacific. Geographical areas were categorized according to Sinka et al. (2012), in which comprehensive distribution data for dominant malaria vectors was gathered and an *Anopheles* global map was created. Ancestral reconstruction was implemented using the ML method [23] available in the APE package [24] of the R programming environment. We also implemented the ancestral geographic range estimation method using the Lagrange software [25].

## Results

**Fig 1:**
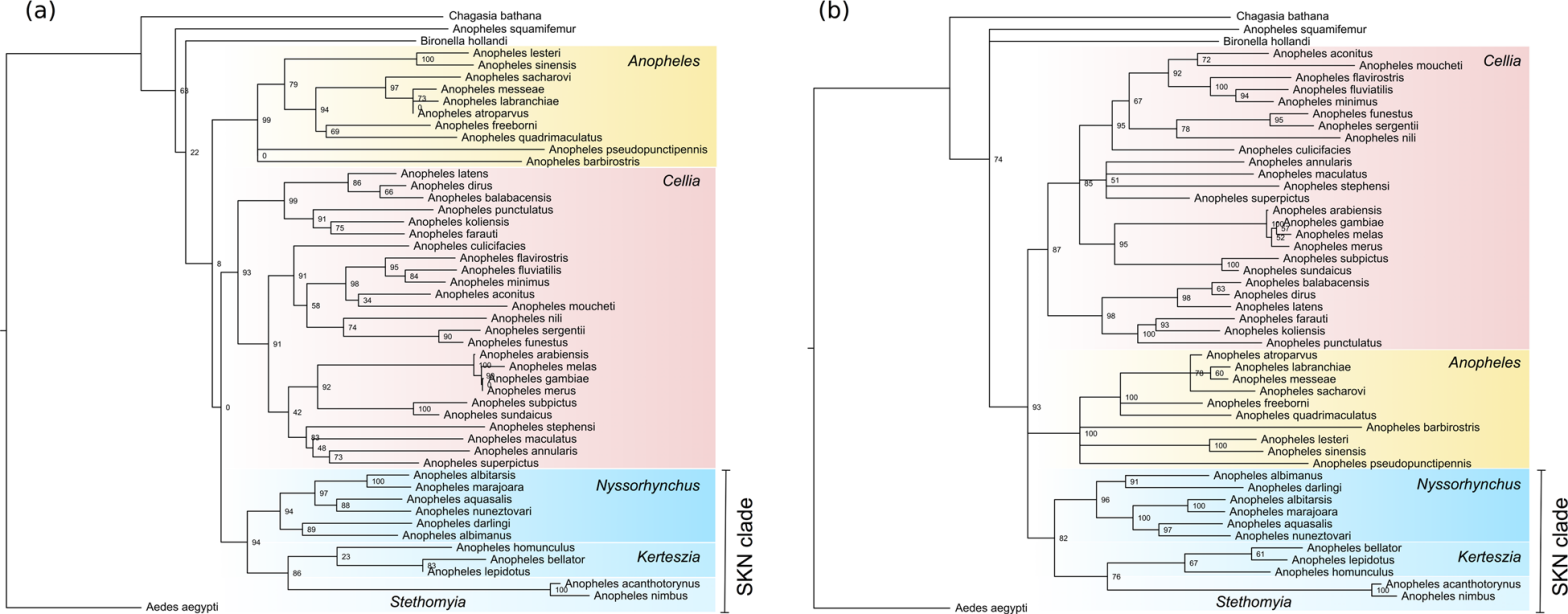
Phylogeny of the *Anopheles* genus. (a) Maximum likelihood tree with aLRT statistical support. (b) Bayesian inference tree with clade posterior probabilities.

Statistical tests performed on our trees suggest a relatively high level of support for our main clades (Fig 1a and 1b). Phylogenetic relationships between major clades were, however, poorly supported. Our results indicate that five subgenera are monophyletic and most of the results are backed with statistical support: *Anopheles* (0.99 aLRT and 100% BP); *Cellia* (0.93 aLRT and 87% BP); *Stethomyia* (1.00 aLRT and 100% BP); *Kerteszia* (0.23 aLRT and 67% BP); and *Nyssorhynchus* (0.94 aLRT and 96% BP; Fig 1a and 1b). The monophyly of subgenus *Lophopodomyia*, however, remains to be tested, because only a single species was included (*A*. *squamifemur*). Data partitioning into nuclear and mitochondrial segments have not altered the topology of *Anopheles* evolution in both Bayesian and ML trees.

As well documented in previous studies, in our tree, we identified genus *Chagasia* as a sister lineage to the clade containing the genera *Bironella* and *Anopheles* [2, 7]. Additionally, a monophyletic classification for genus *Anopheles* was not supported in our trees (Fig 1a and 1b). In the BI tree, *A*. *squamifemur* was retrieved as the sister lineage of the large clade that joined the remaining *Anopheles* diversity plus *Bironella hollandi* (Fig 1a). This result has also been reported previously, and further supports the inclusion of *Bironella* as part of the greater *Anopheles* diversity (morphology Sallum *et al*. 2000; molecular Sallum *et al*. 2002, 2005). On the other hand, in the ML tree, the node was unresolved, joining *A*. *squamifemur*, *B*. *hollandi* and the remaining *Anopheles* spp. in a polytomy.

The paraphyletic status of *Anopheles* in our tree is possibly a result of misplacement of the root. This artifact could occur due to poor outgroup choice or low taxon sampling causing long-branch issues. On the other hand, a comprehensive morphological analysis also indicates the inclusion of *Bironella* lineages within *Anopheles* diversity. Furthermore, we report mixed results as our nodes were not statistically supported, and it would be important to include more sequences of *Bironella* and *Chagasia* to definitively settle this matter.

Due to its large diversity, taxonomists have also assigned the species of *Anopheles* into infrageneric categories such as sections, series, groups, subgroups and complexes. In our dataset, we have included many species from these to enable monophyletic tests of the categories. For instance, subgenera *Anopheles* and *Nyssorhynchus* are divided into sections. We included members of two sections of *Anopheles*, namely, Angusticorn and Laticorn. In this case, the status of these sections is non-resolved due to a polytomy. The polytomic node includes *A*. *pseudopunctipennis* (section Angusticorn), *A*. *barbirotris* (section Laticorn), and the other species of the *Anopheles* subgenus in which diversity is assembled into two monophyletic clades, each with the remaining diversity of each of these two sections. With regard to *Nyssorhynchus*, we also included species assigned to two sections, Albimanus and Argyritarsis, that were not recovered as monophyletic; *A*. *albitarsis* (section Albimanus) grouped with *A*. *marajoara* (section Argyritarsis) with a maximum level of support. The other two species of Albimanus (*A*. *aquasalis* and *A*. *nuneztovari*) also grouped, as did the remaining two species of Argyritarsis (*A*. *darling* and *A*. *albimanus*).

Less inclusive groups include the series in which *Anopheles*, *Cellia* and *Nyssorhynchus* are divided. Our dataset included members of two series of *Anopheles*, series Anopheles (section Angusticorn) and series Myzorhynchus (section Laticorn). Because a single series from each section was included, the same polytomy described above was also observed with regard to the series of *Anopheles*. The genus *Cellia* is represented by members of four series, Myzomyia, Neocellia, Neomyzomyia, and Pyretophorus, none of which are monophyletic. For instance, *A*. *nili* (series Neomyzomyia) grouped within the diversity of the series Myzomyia; the high bootstrap support and the fact that this grouping took place in both topologies indicate that these series are not natural groups. Additionally, *A*. *subpictus* (series Neocellia) and *A*. *sundaicus* (series Pyretophorus) were tightly grouped, rendering their series non-monophyletic groups. Apart from these examples, the remaining diversity of the series grouped for Neocellia (*A*. *annularis*, *A*. *maculatus*, *A*. *superpictus* and *A*. *stephensi*), for Neomyzomyia (*A*. *balabacensis*, *A*. *dirus*, *A*. *farauti*, *A*. *koliensis*, *A*. *latens*, *and A*. *punctulatus*) and for Pyretophorus (*A*. *arabiensis*, *A*. *gambiae*, *A*. *melas*, *A*. *merus*) were so grouped with high support. With regard to *Nyssorhynchus*, four series were included, Albimanus, Albitariss, Argyritarsis and Oswaldoi, but only two included more than a single species. These were series Albitarsis and Oswaldoi, in which both species grouped with high statistical support in our topology.

**Fig 2:**
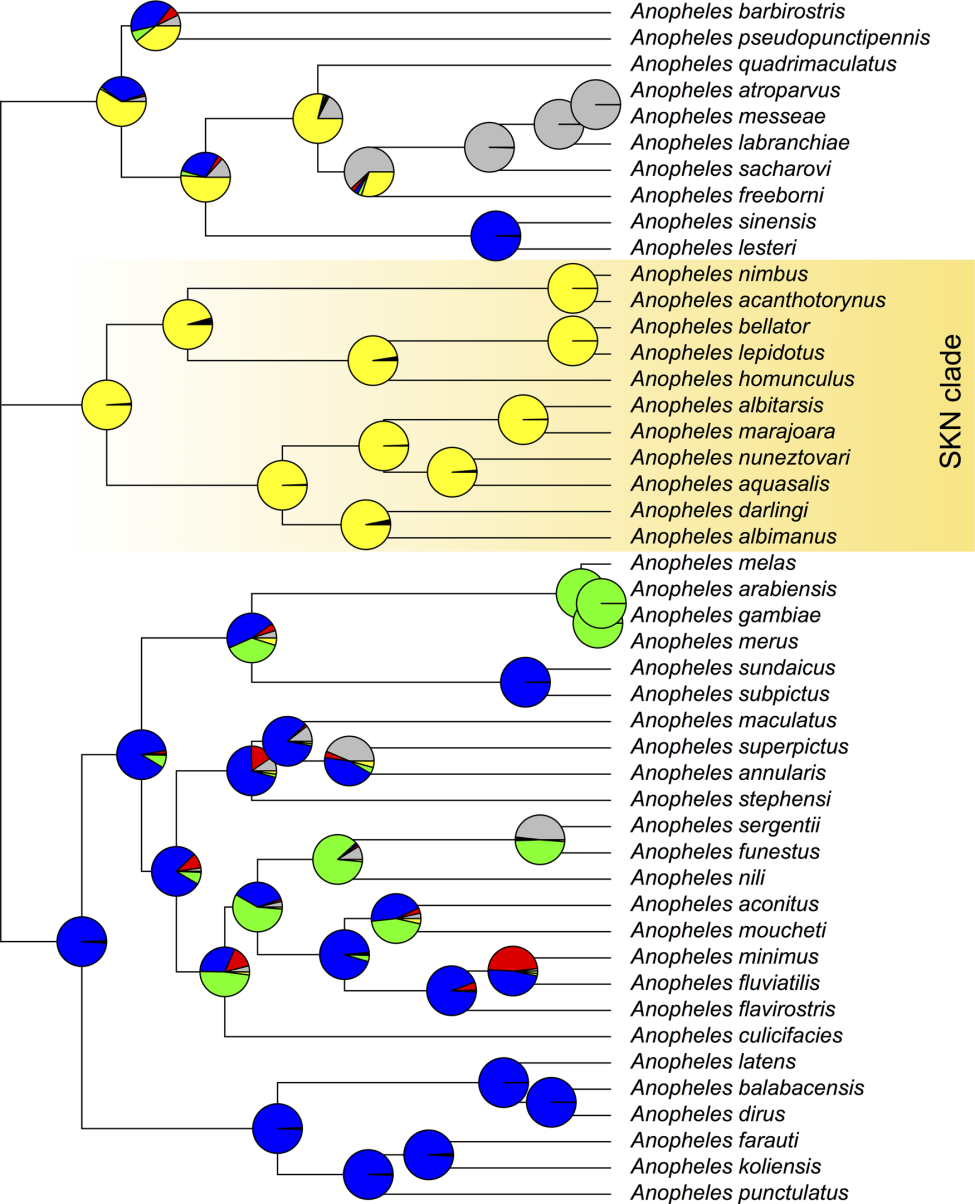
Ancestral area reconstruction conducted using the maximum likelihood method in the APE package. Circles depict the relative probabilities of each region. Color codes are as follows: green—Africa; yellow—Americas; blue—Southeast Asia and the Pacific; gray—Europe plus Middle East; and red—India plus West Asia.

Ancestral area reconstruction presented a higher likelihood that the ancestor of the SKN clade was geographically distributed in the Americas (Fig 2), with 95.2% probability as estimated in Lagrange. An ancestral distribution in Southeast Asia plus the Pacific was favored for the *Cellia* subgenus, whereas the ancestor of the (*A*. *melas*, *A*. *arabiensis*, *A*. *gambiae* and *A*. *merus*) clade was distributed in Africa, with full support in both geographical analyses. Maximum support was also obtained for a Southeast Asia plus the Pacific ancestral distribution for the (*A*. *latens*, *A*. *balabacensis*, *A*. *dirus*, *A*. *farauti*, *A*. *koliensis* and *A*. *punctulatus*) and the (*A*. *sinensis*, *A*.*lesteri*) clades. Finally, the ancestral area of the (*A*. *atroparvus*, *A*. *messeae*, *A*. *labranchiae* and *A*. *sacharovi*) clade was inferred to be India plus West Asia with the highest likelihood. The ancestral geographical distribution of the remaining nodes was not fully resolved.

## Discussion

Our analysis of ancestral geographical distribution is dubious with regard to the *Anopheles* clade, indicating that Neotropic and African distributions in the Early Cretaceous (113 Ma) are equally likely. The timescale inferred here is in agreement with a recent study of 16 *Anopheles* genomes, which also inferred the age of the last common ancestor (LCA) of *Anopheles* at ~100 Ma [26]. Previous studies have estimated more recent ages for the ancestor of *Anopheles*, suggesting a split for *Anopheles* and *Cellia* subgenera in the late Cretaceous, at 93.6 Ma [8] and 81 Ma [21], or even in the Eocene at 43.1 Ma [27]. Moreno et al. [8] and Logue et al. [21] also dated the age of the *Nyssorhynchus* subgenera at 79 Ma and 94 Ma, respectively. Our divergence time for the *Nyssorhynchus* lineage was not comparable with those studies as our analyses grouped these subgenera with *Stethomyia* and *Kerteszia* within the SKN clade.

A comparison between the evolutionary histories of the *Anopheles* genus and *Plasmodium* is meaningful. The phylogeny and timing of the evolution of *Plasmodium* has been the focus of several recent studies [28–31]. It is presumed that the evolution of *Plasmodium* species is strongly associated with host-switching events [29, 32, 33], which poses a major challenge for the elucidation of the *Plasmodium* phylogeny [33]. A total of five *Plasmodium* species are the main causes of malaria in humans: *P*. *falciparum*, *P*. *vivax*, *P*. *ovale*, *P*. *malariae* and *P*. *knowlesi* [34, 35]. Estimates of the divergence times for these species are much younger than those inferred for their *Anopheles* hosts in our analysis, although no consensus on *Plasmodium* divergence times has been reached [31, 33, 36].

In the *Cellia* subgenus, two major clades were clearly recognized; the first lineage separated a clade containing species from Southeast Asia and the Pacific from the remaining species. Additionally, the African group (*A*. *gambiae*, *A*. *merus*, *A*. *melas* and *A*. *arabiensis*) was conspicuously characterized by the small genetic distances between species. Within the *Anopheles* subgenus, ML and BI methods inferred different topological associations. In ML tree, a clade containing the predominantly European species, *A*. *atroparvus*, *A*. *messeae*, *A*. *labranchiae* and *A*. *sacharovi*, was well supported (aLTR 0.97), whereas the same clade presented 78% BP in the Bayesian tree. Moreover, a sister group relationship between *A*. *quadrimaculatus* and *A*. *freeborni* was not recovered in the BI tree. This phylogenetic relationship was recovered in studies that used the ITS2 ribosomal DNA marker [37–39], with minor discrepancies. Moreover, a sister group relationship between *A*. *quadrimaculatus* and *A*. *freeborni* was not recovered in Marinucci et al.’s [37] study.

Phylogenetic relationships within the *Cellia* subgenus are in general agreement with a recent genomic study by Neafsey et al. [26], who also found that the Southeast Asia plus the Pacific (*A*. *dirus + A*. *farauti*) clade consisted of the first lineage to diverge within this subgenus. Differences between our results and that of Neafsey et al. rely on the relationship of major subgenera. Neafsey et al. inferred the (*Nyssorhynchus*, (*Anopheles*, *Cellia*)) relationship, while we could not significantly resolve this higher-level evolutionary relationship within *Anopheles*. In this sense, Krzywinski [2, 3] also recovered the same relationship of Neafsey et al., whereas Sallum [7] estimated a different association, namely, (*Cellia*, (*Nyssorhynchus*, *Anopheles*)).

Specifically, our results are, however, at odds with Kamali et al. and Neafsey at al. with respect to the relationship between *A*. *gambiae*, *A*. *stephensi*, *A*. *funestus* and *A*. *nili*. Both works found a closer evolutionary affinity between *A*. *funestus* and *A*. *stephensi*, with *A*. *gambiae* as sister group, whereas we inferred a (*A*. *funestus* + *A*. *nili*) clade. Both studies, however, favored gene sampling instead of species sampling. Kamali et al. analyzed 49 genes, while Neafsey et al. studied more than 720,000 aminoacid sites. Recent analysis has shown that increasing taxonomic sampling, even with incomplete gene sampling, augments phylogenetic resolution [40]. Therefore, we expect that our analysis, although restricted in the number of loci, gained phylogenetic resolution by the increased sampling of taxa.

It is worth noting that the small genetic distances found in the African group (*A*. *gambiae*, *A*. *merus*, *A*. *melas* and *A*. *arabiensis*) are expected as a result of a complex speciation process permeated with recurrent introgressive hybridization events in the recent evolutionary past, as reported by Fontaine et al. [41]. Controlling and measuring the extent of introgression in phylogenetic reconstruction is not feasible by using a few molecular markers or by including a single representative individual per species. Such thorough investigation is only effective when a large sample of molecular markers and individuals per species is available. Unfortunately, this is not the case of *Anopheles*. With restricted sampling of loci and individuals, it is not feasible to distinguish between phylogenetic discordance caused by stochastic errors, incomplete lineage sorting and introgression. Thus, the extent of the phylogenetic errors due to introgression in *Anopheles* species groups may be unveiled in the future as the number of available genomes increases. Moreover, because of their large population sizes, inference of mosquito species phylogeny is expected to be difficult due to incomplete lineage sorting [42, 43].

The most important topological result of our study, however, is the clade composed of three subgenera of *Anopheles* ((*Stethomyia*, *Kerteszia*), *Nyssorhynchus*). This clade was recovered for the first time in our ML and BI analyses with a relatively high level of support (0.94 aLRT and 82% BP). In this study, we termed this new clade the SKN clade. The association between *Nyssorynchus* + *Kerteszia* has been previously found in molecular [2, 7] and morphological studies [4]. The relationship of the SKN clade with other *Anopheles* subgenera also presents a large discrepancy with other studies. Here, we have found weak support for the sister group relationship between SKN and *Cellia*. Previous studies have placed *Cellia* as a sister-group to the subgenera *Anopheles*, *Lophopodomyia*, *Stethomyia* and the genus *Bironella* [4]; *Cellia* was proposed to be the sister-group of the *Anopheles* subgenus [3], and it has also been associated with the subgenera *Lophopodomyia*, *Nyssorhynchus*, *Kereszia* and genus *Bironella* [7]. The ML topology of this work places the Old-World *Cellia* as a sister-group to the SKN Neotropical clade, although this grouping presented virtually no statistical support (Fig 1 *a*). The discrepancies found between different analyses are likely due to the choice and number of species sampled, which varied significantly among previous studies.

Within SKN, the divergence between subgenera *Stethomyia* and *Kerteszia* was estimated at ca. 70 Ma. Both methods of ancestral reconstruction of geography showed that the LCA of the SKN clade was American with a relative probability of 95.2%, which implies that the early radiation of the SKN subgenera took place in the Americas (Fig 2). The ages of the LCA of subgenera *Anopheles*, *Cellia* and the SKN clade were all inferred to be in the Late Cretaceous at ca. 90 Ma. The geographic distribution of the ancestor of the *Cellia* subgenera was likely in the Southeast Asia and the Pacific region. The ancestral area of the remaining subgenera-level splits could not be resolved with high probability. In conclusion, our results support the concept that the Neotropical subgenera *Stethomyia* + *Kerteszia* + *Nyssorhynchus* comprise a monophyletic group that begun to diversify in the Late Cretaceous. The association of the SKN clade with other *Anopheles* subgenera is unclear. The radiation of the *Cellia* subgenera as well as the *Anopheles* subgenera took place at approximately the same time. Furthermore, although we recovered the monophyly of the *Anopheles* subgenus, our analysis failed to statistically support the monophyly of the *Anopheles* genus.
